# Supplementary material for: The interactome of intact mitochondria by cross-linking mass spectrometry provides evidence for coexisting respiratory supercomplexes
Source: Mol Cell Proteomics. 2017 Dec 8;17(2):216–32. doi: 10.1074/mcp.RA117.000470 (PMC5795388; doi:10.1074/mcp.RA117.000470)
Supplement: Supplemental Data [file supp_RA117.000470_133922_0_supp_23978_fzffwf.zip › spectra_annotation/mito_DR_spectra_annotation/88-1-2-1-7-1.pdf]

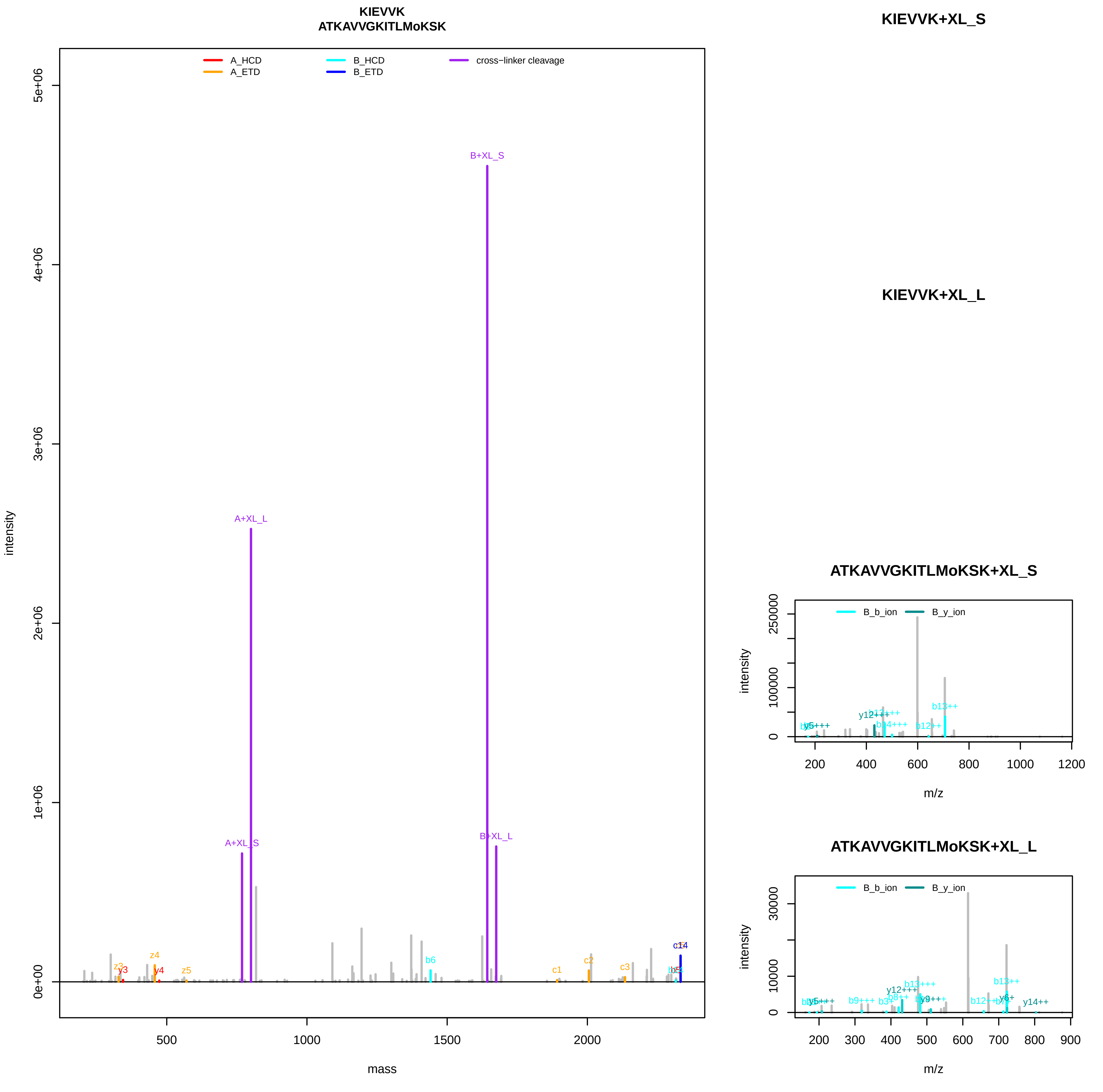
KIEVVK
ATKAVVGKITLMoKSK
A_HCD
A_ETD
B_HCD
B_ETD
cross−linker cleavage
B+XL_S
A+XL_L
A+XL_S
B+XL_L
z3
y3
z4
y4
z5
b6
c1
c2
c3
c14
b5
intensity
0e+00
1e+06
2e+06
3e+06
4e+06
5e+06
500
1000
1500
2000
mass
KIEVVK+XL_S
KIEVVK+XL_L
ATKAVVGKITLMoKSK+XL_S
B_b_ion
B_y_ion
y5+++
y12+++
b13++
b14+++
b12++
intensity
0
100000
250000
200
400
600
800
1000
1200
m/z
ATKAVVGKITLMoKSK+XL_L
B_b_ion
B_y_ion
y5+++
b9+++
b3+
y12+++
b13+++
y9+++
b12++
b13++
y6+
y14++
intensity
0
10000
30000
200
300
400
500
600
700
800
900
m/z
